# Copula directional dependence for inference and statistical analysis of whole‐brain connectivity from fMRI data

**DOI:** 10.1002/brb3.1191

**Published:** 2018-12-27

**Authors:** Namgil Lee, Jong‐Min Kim

**Affiliations:** ^1^ Department of Information Statistics Kangwon National University Chuncheon South Korea; ^2^ Statistics Discipline Division of Sciences and Mathematics University of Minnesota‐Morris Morris Minnesota

**Keywords:** Brodmann area, connectivity, cortex, directional dependence, functional magnetic resonance imaging (fMRI), group analysis

## Abstract

**Introduction:**

Inferring connectivity between brain regions has been raising a lot of attention in recent decades. Copula directional dependence (CDD) is a statistical measure of directed connectivity, which does not require strict assumptions on probability distributions and linearity.

**Methods:**

In this work, CDDs between pairs of local brain areas were estimated based on the fMRI responses of human participants watching a Pixar animation movie. A directed connectivity map of fourteen predefined local areas was obtained for each participant, where the network structure was determined by the strengths of the CDDs. A resampling technique was further applied to determine the statistical significance of the connectivity directions in the networks. In order to demonstrate the effectiveness of the suggested method using CDDs, statistical group analysis was conducted based on graph theoretic measures of the inferred directed networks and CDD intensities. When the 129 fMRI participants were grouped by their age (3–5 year‐old, 7–12 year‐old, adult) and gender (F, M), nonparametric two‐way analysis of variance (ANOVA) results could identify which cortical regions and connectivity structures correlated with the two physiological factors.

**Results:**

Especially, we could identify that (a) graph centrality measures of the frontal eye fields (FEF), the inferior temporal gyrus (ITG), and the temporopolar area (TP) were significantly affected by aging, (b) CDD intensities between FEF and the primary motor cortex (M1) and between ITG and TP were highly significantly affected by aging, and (c) CDDs between M1 and the anterior prefrontal cortex (aPFC) were highly significantly affected by gender.

**Software:**

The R source code for fMRI data preprocessing, estimation of directional dependences, network visualization, and statistical analyses are available at https://github.com/namgillee/ CDDforFMRI.

## 1 INTRODUCTION

Identifying brain connectivity engaged in various cognitive tasks is an important research topic in neuroscience. It has long been known that the brain can be segregated anatomically into several separate regions, which may also act as functionally distinguishable units (Brodmann, 1909, 2006). On the other hand, brain connectivity concerns the integration of such segregated regions within a nerve system at several levels of scales, for example, microscale, mesoscale, and macroscale (Sporns, 2010). The concept of brain connectivity can be different and inconsistent across studies so it should be used with caution (Horwitz, 2003). It is commonly accepted that there are three types of brain connectivity, which are structural connectivity, functional connectivity, and effective connectivity (Friston, 1994). Among the three types, functional connectivity is defined as statistical dependence between spatially remote neurophysiological events, and effective connectivity refers to the influence one neural system exerts over another, while their operational definitions can vary depending on the model we use (Friston, 1994).

The main purpose of this research was to identify and validate functional connectivity obtained by using a statistical measure of directional dependence which is called the copula directional dependence (CDD) (Kim & Hwang, 2017, 2018; Sungur, 2006) and by using human brain functional magnetic resonance imaging (fMRI) data. One advantage of the CDD is that the CDD measure can be applied to non‐normal distributions and nonlinear relationships. Another advantage is that, whereas most of the functional connectivity measures such as correlations and mutual information are undirected (Wang et al., 2014), the CDD can produce bidirectional connectivity which identifies statistical influence from one brain region to another.

The directional connectivity inferred by CDDs is different from and has some advantages over the popular effective connectivity measures as follows.

### 1.1 Advantages over dynamical systems models

Although both the CDD and effective connectivity measures produce directed connectivity, the CDD is distinguished from effective connectivity in that it does not rely on dynamical systems modeling of time‐dependent brain activity. Research on inferring effective connectivity using fMRI data mostly focuses on stochastic dynamical system modeling (Stephan & Friston, 2010). For example, the dynamic causal models (DCMs) incorporate a set of partial differential equations of time‐dependent state variables representing the postsynaptic membrane potentials of neural systems (Friston, Harrison, & Penny, 2003; Stephan et al., 2007). For another example, multivariate/vector autoregressive models (MAR/VAR) have been applied for discrete‐time stochastic dynamical system modeling of fMRI (Goebel, Roebroeck, Kim, & Formisano, 2003; Harrison, Penny, & Friston, 2003; Roebroeck, Formisano, & Goebel, 2005). Besides stochastic dynamical system modeling or Granger causality (GC), several other measures of directed connectivity are often used; see, for example, transfer entropy (TE) (Vicente, Wibral, Lindner, & Pipa, 2011), directed information (DI) (Wang, Alahmadi, Zhu, & Li, 2016), and convergent cross mapping (CCM) (Sugihara et al., 2012).

In principle, identifying which variables are causes and which are effects is not a trivial task. In order to avoid the identification problem, dynamical system modeling approaches include a temporal delay of *all* the relevant variables in the system equations (Granger, 1969; Sims, 1980), which inevitably increases model complexity. Typically, a suitable model selection procedure should follow the dynamical system modeling to reduce model complexity and to prune spurious cause–effect relationships (Lee, Kim, Park, & Kim, 2016; Stephan & Friston, 2010). Whereas such model selection procedures usually include assumptions on specific probability distributions, the suggested CDD does not require strict distribution assumptions.

In addition, we note that the temporal resolution of fMRI is relatively low, which can cause a problem in the decision on cause–effect relationships in dynamical systems modeling. Specifically, the temporal resolution of fMRI is as low as two or three‐seconds per scan, whereas the time taken for information transfer in neural systems associated with cognitive functions is roughly 250 to 500 milliseconds when measured as event‐related potentials (ERPs) by electroencephalography (EEG) (Polich, 2007). Such low sampling rates can result in reversed cause–effect relationships in dynamical system models. See Section *Simulation Evaluation* for an example using VAR models.

### 1.2 Advantages over graphical causal models

On the other hand, besides the popular dynamical systems modeling approaches for causal inference, there have been various approaches to defining and modeling causal relations from observational sample data or experimental data in statistics and machine‐learning communities, which are often called graphical causal models (Spirtes, 2010). Graphical causal models include causal Bayesian networks and structural equation models (SEMs).

In principle, the graphical causal modeling approaches select directed graph structures which satisfy the so‐called Markov assumption and faithfulness assumption (Pearl, 2000; Spirtes, 2010; Spirtes, Glymour, & Scheines, 1993). Roughly speaking, a directed graph satisfies the Markov assumption if the observed distribution satisfies the conditional independences imposed by the graph structure; it satisfies the faithfulness assumption if every conditional independence in the observed distribution is entailed by the graph structure. However, even if the Markov and faithfulness assumptions are imposed, the true causal model cannot be determined among several directed acyclic graphs that possess the same independence structures, which is called the Markov equivalence class. In particular, the directed graphs *X* → *Y* and *Y* → *X* for two variables cannot be distinguished.

An advantage of the CDD over the graphical causal models is that the CDD produces bidirectional connectivity between two variables, that is, for a pair $\left(X,Y\right)$ of variables of interest, a CDD measure from *X* to *Y* and one from *Y* to X are produced. Such bidirectional connectivity enables us to compare the relative strengths of the directional dependences and to determine the statistical significance of inferred directionalities.

### 1.3 Copula directional dependence and beta regression in literatures

Numerous studies have suggested regression‐based approaches for the determination of cause and effect for two time‐independent variables, say *X* and *Y*. These approaches investigate asymmetry in the distribution of the two variables by comparing the regression models in alternate directions. Kano and Shimizu (2003) and Shimizu, Hoyer, Hyvärinen, and Kerminen (2006) suggested a method for a linear non‐Gaussian acyclic model (LiNGAM), which can be written as

$$Y=f\left(X\right)+N,$$

where *f* is a linear function and *N* is a non‐Gaussian independent noise. Hoyer, Janzing, Mooij, Peters, and Schölkopf (2009) and Mooij, Janzing, Peters, and Schölkopf (2009) generalized the LiNGAM to nonlinear additive noise models (ANMs). Zhang and Hyvärinen (2009) introduced a generalization of ANMs as post‐nonlinear causal models (PNL).

A common criterion to distinguish between cause and effect for these methods is that whenever a regression model with independent noise can be fit in only one direction, one infers that direction to be the causal direction. Janzing and Steudel (2010) investigated theoretical properties of the criterion by using the concept of Kolmogorov complexity. The information‐geometric approach for causal inference (IGCI) was proposed based on a certain independence condition between the conditional distribution and the marginal distribution in information space (Janzing et al., 2012). The causal inference with unsupervised inverse regression (CURE) method proposed by Sgouritsa, Janzing, Hennig, and Schölkopf (2015) uses the same principle of independence in an unsupervised manner.

Recently, it was proven that the criterion for causal inference under the independent additive noise models can be reduced to a much simpler form based on regression errors (Blöbaum, Janzing, Washio, Shimizu, & Schölkopf, 2018). Simply speaking, the mean‐squared error (MSE) of prediction in the correct cause–effect direction is smaller than that in the other direction under mild independence assumptions. Moreover, the simplified criterion can yield an inference method with a significantly lower computational cost than previously known methods (Blöbaum et al., 2018). However, simple criteria such as the MSE cannot be used unless the cause and effect variables are properly scaled. Since most of the real world data are non‐normally distributed with various scales, it is important to propose a simple criterion that does not sensitively depend on marginal distributions of the cause and effect variables.

A copula is a multivariate function which provides flexible and effective ways for describing statistical dependencies, especially between non‐normal random variables. It was shown that any joint distribution function can be expressed as a copula function which combines one‐dimensional marginal distributions (Sklar, 1973). Accordingly, an attractive property of copulas is that statistical dependence structures can be modeled by choosing a copula function independently of the marginal distributions. Moreover, the dependence structure determined by a copula function is invariant under one‐to‐one continuous transformations of each variable. In addition, a normal distribution assumption or linearity is not required for copula‐based dependence modeling. Copulas have been widely applied in many fields such as macroeconomics and finance (Cherubini, Luciano, & Vecchiato, 2004; Cherubini, Mulinacci, Gobbi, & Romagnoli, 2012), genetics (Kim et al., 2008; Li, Boehnke, Abecasis, & Song, 2006), and neuroscience (Hu & Liang, 2014; Ince et al., 2015, 2017).

Regression models using copulas have been studied widely in various ways. Especially, the Gaussian copula regression method can conveniently express dependence in the form of a correlation matrix, and the likelihood inference for continuous responses can be carried out efficiently (Masarotto & Varin, 2012; Pitt, Chan, & Kohn, 2006; Song, 2000; Song, Li, & Yuan, 2009).

However, the standard Gaussian copula regression method has a limitation in that it cannot deal with asymmetric dependence. Directional dependence refers to asymmetric dependence between variables. Initially, the concept of directional dependence was studied under linear regression models regardless of copulas (Dodge & Rousson, 2000, 2001). Its concept was defined and studied in regression settings using copulas by Sungur (2006).

Recently, a new directional dependence measure based on the beta regression model of Guolo and Varin (2014) and copula regression was proposed in Kim and Hwang (2017, 2018). A beta regression is a regression model for continuous responses taking values in unit intervals such as rates and proportions (Ferrari & Cribari‐Neto, 2004). The regression model is called the beta regression model because it uses beta distribution for modeling the response variables. Due to the flexibility of the beta distribution, it can handle a wide variety of distributions with various shapes and asymmetries, and the beta regression model can deal with heteroscedasticity and asymmetry in regression problems. Several variants of the beta regression model have been proposed such as beta regression with nonlinearity and variable dispersion (Cribari‐Neto & Zeileis, 2010), Bayesian approaches (Casarin, Leisen, Molina, & ter Horst, 2015; Casarin, Valle, & Leisen, 2012; Figueroa‐Zuniga, Arellano‐Valle, & Ferrari, 2013), and zero‐or‐one inflated beta regressions (Ospina & Ferrari, 2012). A beta regression model for bounded time series was proposed in Guolo and Varin (2014), where the serial correlation is addressed by the Gaussian copula with a correlation matrix of the stationary autoregressive moving average (ARMA) process.

In this paper, we apply the CDDs proposed in Kim and Hwang (2017, 2018) to the inference of brain connectivity and statistical group analysis from fMRI data. The main advantage of the suggested method is that the non‐normality and nonlinearity in the distributions for fMRI data can be effectively addressed by the CDD measures. The experimental results based on simulated data and real fMRI data demonstrate that the estimated CDD connectivity measures could produce biologically plausible networks of brain regions relevant to participants’ physiological factors. We remark that previous studies on copula‐based methods for brain imaging data analysis focused either on inferring effective connectivity with Granger causality using time‐dependent variables (Hu & Liang, 2014) or on deriving symmetric connectivity measures such as mutual information (Ince et al., 2015, 2017). In contrast, the CDD measure suggested in this study infers asymmetric connectivity based on time‐independent variables with serial correlation removed.

### 1.4 Directional dependence using beta regression

#### 1.4.1 Directional dependence by copula regression

A copula is a multidimensional distribution function with uniformly distributed marginal distributions. In this paper, we focus on two‐dimensional copulas. A joint cumulative distribution function (CDF), $F_{\mathrm{XY}}\left(x,y\right)=\mathrm{Pr}\left(X\leq x,\;Y\leq y\right)$, can be represented by the composition of a bivariate function, $C\left(u,v\right)$, with the two marginal CDFs $F_{X}\left(x\right)=\mathrm{Pr}\left(X\leq x\right)$ and $F_{Y}\left(y\right)=\mathrm{Pr}\left(Y\leq y\right)\;$ (Sklar, 1973) as

$$F_{\mathrm{XY}}\left(x,y\right)=C\left(F_{X}\left(x\right),F_{Y}\left(y\right)\right),$$

where $C\left(u,v\right)$ is called the copula. We can see that the copula $C\left(u,v\right)$ determines the dependency structure between two random variables *X* and *Y*. Note that $U=F_{X}\left(X\right)$ and $V=F_{Y}\left(Y\right)$ have uniform distribution on $\left[0,1\right]$. Hence, the copula is independent of marginal distributions and any one‐to‐one transformations of them.

In general, directional dependence can be defined in terms of regression using a copula function (Sungur, 2006). Let $\left(U,V\right)$ denote a pair of random variables whose marginal distributions have uniform distribution on $\left[0,1\right]$ and the joint distribution is a copula function $F_{\mathrm{UV}}\left(u,v\right)=C\left(u,v\right)$. Let $C_{u}\left(v\right)$ denote the conditional distribution of *V* given *U* = *u* as $C_{u}\left(v\right)\equiv P\left(V\leq v|\;U=u\right)=\partial C\left(u,v\right)/\partial u.$ The copula regression function of *U* on *V* is the conditional expectation of *V* given *U* = *u*, which can be expressed by the copula as

$$r_{V|U}\left(u\right)\equiv E\left[V|\;U=u\right]=1-\int_{0}^{1}C_{u}\left(v\right)dv.$$

The directional dependence from *U* to *V* is defined by using the copula regression function on *V* as

(1) $$\rho_{U\to V}^{2}\equiv \frac{\text{Var}\left(r_{V|U}\left(U\right)\right)}{\text{Var}\left(V\right)}=\frac{E[\left(r_{V|U}\left(U\right)-0.5{)}^{2}\right]}{1/12}=12E\left[(r_{V|U}\left(U\right){)}^{2}\right]-3,$$

which can be interpreted as the proportion of total variance of *V* that has been explained by the copula regression function $r_{V|U}\left(u\right)$. In the same way, the directional dependence from *V* to *U* is defined by the proportion of total variance of *U* that has been explained by the copula regression function $r_{U|V}\left(v\right)$ as

(2) $$\rho_{V\to U}^{2}\equiv \frac{\text{Var}\left(r_{U|V}\left(V\right)\right)}{\text{Var}\left(U\right)}=\frac{E[\left(r_{U|V}\left(V\right)-0.5{)}^{2}\right]}{1/12}=12E\left[(r_{U|V}\left(V\right){)}^{2}\right]-3.$$

Note that if *U* and *V* are independent, then $C\left(u,v\right)=uv$ and $r_{V|U}\left(u\right)=r_{U|V}\left(v\right)=0.5$, which implies that the directional dependences in Equations (1) and (2) can be interpreted as measures of deviations from independence. Moreover, we can compare the two‐directional dependences to identify which copula regression can explain more variances and has higher prediction capabilities.

#### 1.4.2 Beta regression

To model directional dependences by copula, it is necessary to determine an appropriate and efficient parametric form of the copula regression function for the inference of a dependence structure from data. In beta regression (Ferrari & Cribari‐Neto, 2004; Guolo & Varin, 2014), a response variable *V*_*t*_ given *U*_*t*_ = *u*_*t*_ has a beta distribution, $\mathrm{Beta}\left(\mu_{t},\kappa_{t}\right)$, with the mean parameter 0 < *μ*_*t*_ < 1 and the precision parameter *κ*_*t*_ > 0. The density function of $V_{t}|\;U_{t}=u_{t}$ is written as

$$f\left(v_{t}|\;\mu_{t},\kappa_{t}\right)=\frac{\Gamma \left(\kappa_{t}\right)}{\Gamma \left(\mu_{t}\kappa_{t}\right)\Gamma \left(\left(1-\mu_{t}\right)\kappa_{t}\right)}v_{t}^{\mu_{t}\kappa_{t}-1}{\left(1-v_{t}\right)}^{\left(1-\mu_{t}\right)\kappa_{t}-1},$$

where $\Gamma \left(\cdot \right)$ is the gamma function. The mean parameter *μ*_*t*_ is linked with the covariate *u*_*t*_ by the logit function as

(3) $$\mathrm{logit}\left(\mu_{t}\right)=u_{t}\beta_{1}+\beta_{0}.$$

By using the beta distribution, we can model a wide variety of distributions with various locations and shapes over bounded intervals. The parameters *β*_0_ and *β*_1_ can be estimated based on maximum‐likelihood approaches (Guolo & Varin, 2014; Masarotto & Varin, 2012). The serial correlation which was not explained by the beta regression model in (3) can be modeled by a marginal regression model developed in Guolo and Varin (2014). See Guolo and Varin (2014) for more technical details.

### 1.5 Statistical significance of connectivity direction based on bootstrap confidence intervals

In studies of brain connectivity, an important issue is to determine the direction of each connection between brain regions. Since the suggested CDD connectivity network is bidirectional, that is, connectivity measures exist in both directions, the connectivity direction is defined as the direction of the stronger CDD measure. Therefore, the direction is determined based on the sign of the difference,

$$\Delta \rho_{U,V}^{2}=\rho_{U\to V}^{2}-\rho_{V\to U}^{2},$$

for a pair of brain regions $\left(U,V\right)$.

In this study, we applied a basic bootstrap resampling technique in order to compute a 95% confidence interval for the difference $\Delta \rho_{U,V}^{2}$, which is denoted by

$$\left[LB\left(\Delta \rho^{2}\right),\;UB\left(\Delta \rho^{2}\right)\right],$$

where $LB\left(\Delta \rho^{2}\right)$ and $UB\left(\Delta \rho^{2}\right)$ are the lower and upper limits of the 95% confidence interval. Specifically, we used the ordinary nonparametric bootstrap with 100 bootstrap replicates and produced basic bootstrap confidence intervals.

### 1.6 Simulated experiments

We conducted two different types of simulated experiments in order to validate the performance of the proposed CDD measure for inferring causal relationships.

### 1.7 Sensitivity analysis based on simulated fMRI data from asymmetric copula distribution

**Figure 1:**
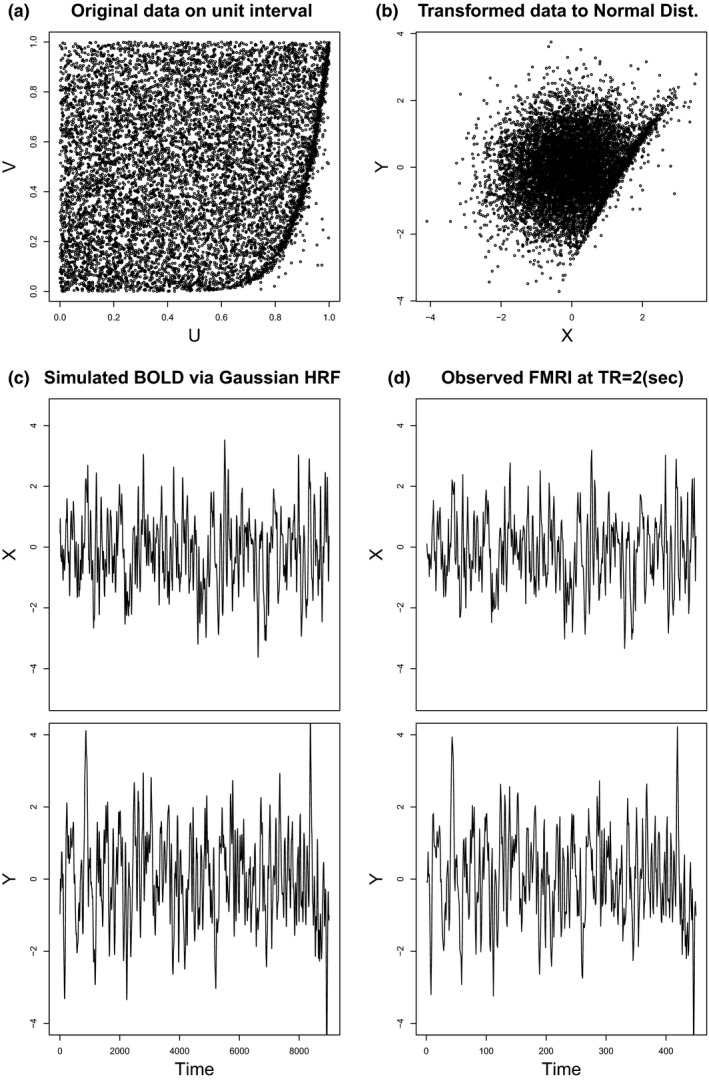
Illustration of the data generation procedure for the simulated fMRI data. (a) The original bivariate data generated by the predefined asymmetric copula distribution on the square region ${\left[0,1\right]}^{2}$ with *β* = 0.1 and *T* = 15, that is, *n* = 60*T*/*a* = 9000. (b) The transformed data to the standard normal distribution. (c) The simulated bivariate BOLD time series data after the convolution with the Gaussian HRF with the FWHM of 4 s. (d) The simulated fMRI data subsampled at every TR = 2 s

Note that a cause–effect relationship between two variables *X* and *Y* can be formed when their joint distribution is not symmetric. In this section, simulated resting‐state fMRI (RS‐fMRI) data of two regions‐of‐interest (ROIs) were generated from an asymmetric distribution based on R packages copBasic and neuRosim as follows. The data generation procedure is also described in Figure 1.

- An asymmetric copula distribution function on unit square ${\left[0,1\right]}^{2}$ was constructed by combining two symmetric copulas as (Durante, 2009)
  (4) $$C_{\alpha ,\beta }\left(u,v\right)=A_{\theta_{1}}\left(u^{\alpha },v^{\beta }\right)A_{\theta_{2}}\left(u^{\overset{¯}{\alpha }},v^{\overset{¯}{\beta }}\right),$$
  where $\alpha ,\;\beta \in \left(0,1\right)$, $\overset{¯}{\alpha }=1-\alpha$, $\overset{¯}{\beta }=1-\beta$, and $A_{\theta }\left(u,v\right)$ is a Plackett copula with parameter *θ* > 0 (Plackett, 1965). The Plackett copula is available as the function PLACKETcop() in the R package copBasic. For simulation, we set *θ*_1_ = 5000, *θ*_2_ = 5, *α* = 1 ‐ *β*, and β=0.1,0.2,…,0.5. We remark that the copula $C_{\alpha ,\beta }\left(u,v\right)$ with *α* = *β* is symmetric.
- The simulated RS‐fMRI data are assumed to have a time length of *T* minutes, *T* = 5, 10, …, 30. With an accuracy parameter of *a* = 0.1, we generated the bivariate data set $\left(u_{i},v_{i}\right),\;i=1,\ldots ,n$, of length *n* = *T* * 60/*a* from the asymmetric copula in (4). See Figure 1a for an illustration of the generated data for *β* = 0.1 and *T* = 15.
- The data set was transformed to have the standard normal distribution as its marginal distribution by the probability integral transform as $x_{i}=\Phi^{-1}\left(u_{i}\right),\;y_{i}=\Phi^{-1}\left(v_{i}\right)$, where *Φ* is the standard normal CDF. See Figure 1b.
- The Gaussian hemodynamic response function (HRF) with the Full Width Half Maximum (FWHM) of 4 (s) was convolved with each of the time series variables *x*_*i*_ and *y*_*i*_ to yield the simulated BOLD responses. See Figure 1c.
- The fMRI measurements at the repetition time (TR) of 2 (s) were subsampled from the simulated BOLD responses. See Figure 1d.

**Figure 2:**
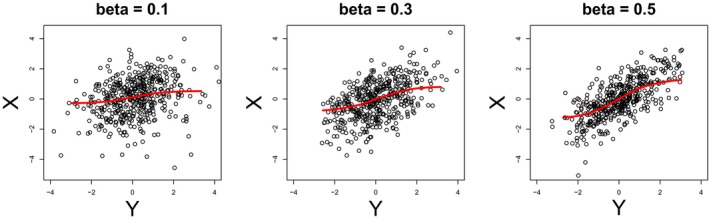
Scatter plots of the simulated fMRI data for β=0.1,0.3,0.5 and *T* = 15, together with the regression curves fitted by the proposed method

Figure 2 illustrates the scatter plots of the simulated fMRI data for β=0.1,0.3,0.5 and *T* = 15, together with the regression curves fitted by the proposed method. The regression curves are nonlinear as described in Equation 3. In addition, we can find that the correlation or slope increased as *β* increased.

The sensitivity of the proposed method was analyzed based on the bias, standard deviation (SD), and root‐mean‐squared error (RMSE) for the estimate of the difference, $\Delta \rho_{X,Y}^{2}$. Note that the difference, $\Delta \rho_{X,Y}^{2}$, is a measure of causal direction. The three sensitivity measures can be computed through the bootstrap resampling procedure described in the previous section. The results of the sensitivity analysis are summarized in Table 1. We can find that the bias was relatively smaller than the SD and RMSE. The RMSE tended to increase as the value of *β* increased. Note that as the value of *β* increases, the correlation between the two variables increases (see, e.g., Figure 2) but the asymmetry of the sampling distribution decreases because the difference between *β* and *α* = 1 ‐ *β* decreases.

**Table 1:** Sensitivity analysis for the proposed CDD measure based on simulated fMRI data generated from asymmetric distributions

*β*		*T* = 5	*T* = 10	*T* = 15	*T* = 20	*T* = 25	*T* = 30
0.1	Bias	−0.0005	−0.0004	0.0005	0.0009	−0.0003	−0.0004
	SD	0.0036	0.0091	0.0044	0.0039	0.0035	0.0025
	RMSE	0.0037	0.0091	0.0044	0.0040	0.0035	0.0026
0.2	Bias	0.0021	−0.0003	−0.0006	0.0003	−0.0001	0.0000
	SD	0.0167	0.0139	0.0063	0.0054	0.0063	0.0077
	RMSE	0.0168	0.0139	0.0063	0.0054	0.0063	0.0077
0.3	Bias	−0.0024	0.0010	−0.0021	0.0014	−0.0001	0.0000
	SD	0.0200	0.0134	0.0125	0.0104	0.0104	0.0085
	RMSE	0.0202	0.0134	0.0126	0.0105	0.0104	0.0085
0.4	Bias	−0.0022	−0.0002	−0.0001	−0.0009	−0.0015	−0.0016
	SD	0.0178	0.0155	0.0115	0.0120	0.0096	0.0100
	RMSE	0.0179	0.0155	0.0115	0.0120	0.0097	0.0101
0.5	Bias	−0.0033	0.0014	0.0025	0.0001	0.0015	−0.0003
	SD	0.0281	0.0181	0.0153	0.0119	0.0107	0.0111
	RMSE	0.0282	0.0181	0.0155	0.0119	0.0108	0.0111

**Figure 3:**
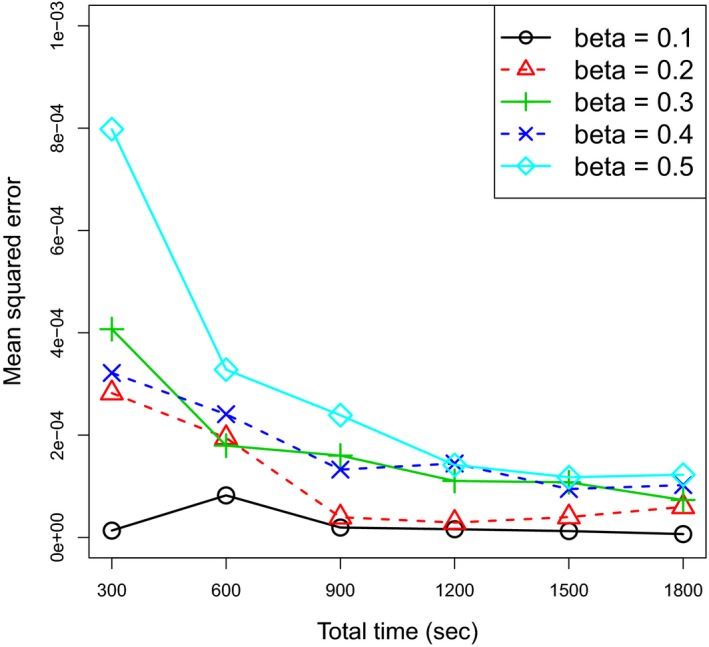
The root‐mean‐squared error (RMSE) values for various values of the model parameter *β* and the data length *T* for the simulated fMRI data in the simulated experiments

Figure 3 shows the RMSE values for all *β* and *T* values. We can clearly find that the RMSE values tended to decrease as *T* increased.

### 1.8 An example of a VAR process with reversed cause–effect relationships

In this section, we present an example which indicates that if the data points are measured with a low sampling rate, then inferences for effective connectivity using dynamical system models can lead to a reversed direction of connectivity.

Assume that a neural system of three brain regions follows a VAR model of order 1 expressed by

(5) $$y_{t}={\mathrm{Ay}}_{t-1}+\epsilon_{t},\;t=1,2,\ldots$$

where $y_{t}={\left(y_{t1},y_{t2},y_{t3}\right)}^{⊤}$ and **ϵ**_*t*_ = (ϵ_*t*1_, ϵ_*t*2_, ϵ_*t*3_) ^⊤^ are vectors of brain signals and white noises. Let the coefficient matrix **A** be given by

$$A=\left(\begin{array}{ccc} 0 & 0.5 & 0\\ 0 & 0 & 0.5\\ 0.5 & 0 & 0 \end{array}\right).$$

The cause–effect relationships between the three brain regions, denoted by “[1],” “[2],” and “[3],” are determined by the nonzero coefficients in **A**, which can be summarized as

$$\left[2\right]\to \left[1\right],\;\;\left[3\right]\to \left[2\right],\;\;\left[1\right]\to \left[3\right].$$

Note that there are no bidirectional relationships, that is, $\left[i\right]\to \left[j\right]$ and $\left[j\right]\to \left[i\right]$ for some *i* and *j*.

Suppose that the data are measured with a low sampling rate as in fMRI experiments, say *L* ≥ 2 times lower sampling rate. In this case, from the original model equation in Equation 5, the measured time series data, $y_{\mathrm{Lt}}\;\left(t=1,2,\ldots \right)$, can be expressed by a VAR model of order 1 as

(6) $$y_{\mathrm{Lt}}={\mathrm{Ay}}_{Lt-1}+\epsilon_{\mathrm{Lt}}=A\left({\mathrm{Ay}}_{Lt-2}+\epsilon_{Lt-1}\right)+\epsilon_{\mathrm{Lt}}=\cdots =A^{L}y_{L(t-1)}+\eta_{\mathrm{Lt}},$$

where $\eta_{\mathrm{Lt}}=\sum_{i=1}^{L}A^{L-i}\epsilon_{L(t-1)+i}$ is a white noise that is independent of $y_{L\left(t-1\right)}$. When L=2,5,8,…, the coefficient matrix **A** ^*L*^ can be expressed by

$$A^{L}=\left(\begin{array}{ccc} 0 & 0 & 0.5^{L}\\ 0.5^{L} & 0 & 0\\ 0 & 0.5^{L} & 0 \end{array}\right),$$

and the corresponding cause–effect relationships between the three brain regions can be expressed by

$$\left[1\right]\to \left[2\right],\;\;\left[2\right]\to \left[3\right],\;\;\left[3\right]\to \left[1\right].$$

Note that all three of the cause–effect relationships are reversed compared to the original ones derived from **A**.

For an evaluation of the performance of the proposed method, trivariate time series data of length *n* = 10000 were generated from the VAR model in Equation 5. The white noise process was **ϵ**_*t*_ = (ϵ_*t*1_, ϵ_*t*2_, ϵ_*t*3_) ^⊤^ generated from three different types of distributions as follows:

1. Model 1. Normally distributed independent noise: The three noise components were independently and normally distributed as ϵ_*t*1_, ϵ_*t*2_, ϵ_*t*3_ ˜i.i.d. N(0, 1).
2. Model 2. Symmetrically dependent noise: The first two components were generated from a bivariate normal distribution as (ϵ_*t*1_, ϵ_*t*2_) ˜i.i.d. N_2_(**0**,** R**), **R** = (*r*_*ij*_)with the variances *r*_11_ = *r*_22_ = 1 and the correlations *r*_12_ = *r*_21_ = 0.56. And ϵ_*t*3_ ˜i.i.d. N(0,1).
3. Model 3. Asymmetrically dependent noise: The first two components were generated from the copula distribution described in Equation 4, that is, (ϵ_*t*1_, ϵ_*t*2_) ˜i.i.d. *C*_*α,β*_ (*u, v*) with α=0.7,β=0.3.

Next, the generated time series data were subsampled at the rate of 1 in *L* = 5, so that the subsampled data follow the lagged VAR model in Equation 6.

**Figure 4:**
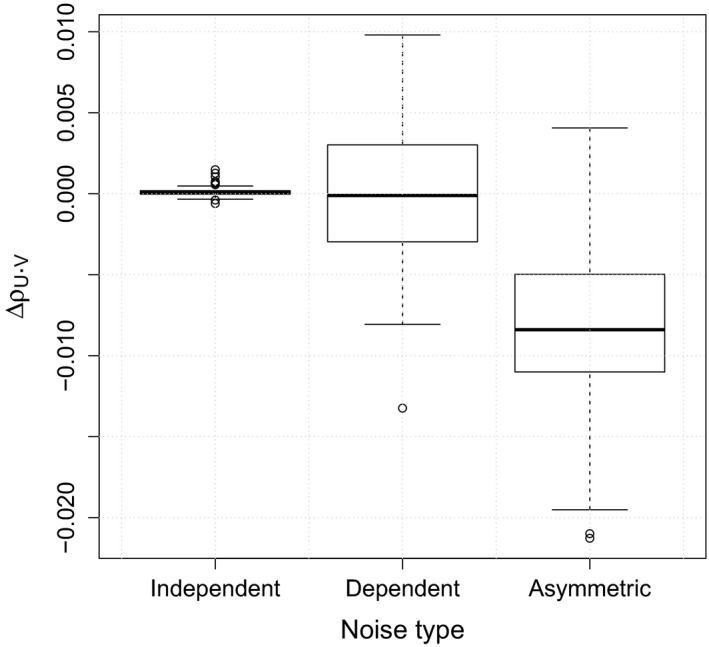
Boxplots of the differences, $\Delta \rho_{y_{1},y_{2}}^{2}$, obtained by the bootstrap resampling procedure for the three types of noise for the three‐dimensional VAR models in the simulated experiments. The negative sign $\Delta \rho_{y_{1},y_{2}}^{2}<0$ implies that the determined direction is *y*_2_ → *y*_1_

The performances of the proposed method were evaluated based on the CDDs estimated for the first two variables, $\left(y_{t1},y_{t2}\right)$, and the cause–effect directions determined by the difference of the CDDs, $\Delta \rho_{y_{1},y_{2}}^{2}$. The experimental results are summarized in Table 2. In the table, the *p*‐value considers the test of the null hypothesis that $\Delta \rho_{y_{1},y_{2}}^{2}=0$, and the relative RMSE is defined by the RMSE divided by the absolute value of $\Delta \rho_{y_{1},y_{2}}^{2}.\;$ In the table, we can see that the value of $\Delta \rho_{y_{1},\;y_{2}}^{2}$ was negative only for the case of Model 3, which implies that the asymmetric distribution of the noise process affected the determination of the directionality rather than the nonzero values of the VAR coefficients. For Model 1, the correlation between *y*_1_ and *y*_2_ was close to zero, which resulted in small values of CDDs, their difference, and *p*‐value. In addition, the relative RMSE value was smaller than 1 only for Model 3, which implies that the accuracy was higher than in the other models. Figure 4 shows boxplots of the differences, $\Delta \rho_{y_{1},\;y_{2}}^{2}$, obtained by the bootstrap resampling procedure for the three types of noise. We can clearly see that the independent noise and dependent noise models could not determine the correct direction between *y*_1_ and *y*_2_, but the asymmetric noise model could determine the direction *y*_2_ → *y*_1_ based on $\Delta \rho_{y_{1},\;y_{2}}^{2}<0$.

**Table 2:** Performances of the proposed CDD method for three‐dimensional VAR models with three types of noise processes

	Model 1 independent noise	Model 2 dependent noise	Model 3 asymmetric noise
cor(ε_1_, ε_2_)	0.00	0.56	0.56
$\mathrm{cor}\left(y_{1},y_{2}\right)$	−0.01	0.42	0.42
$\rho_{y_{1}\to y_{2}}^{2}$	0.000	0.160	0.153
$\rho_{y_{2}\to y_{1}}^{2}$	0.000	0.160	0.161
$\Delta \rho_{y_{1},\;y_{2}}^{2}$	0.0001	0.0001	−**0.0087**
$LB\left(\Delta \rho^{2}\right)$	0.0001	−0.0008	−0.0093
$UB\left(\Delta \rho^{2}\right)$	0.0002	0.0009	−0.0074
*p*‐value	0.0002**	0.9420	0.0000**
Bias	0.0000	−0.0002	0.0004
SD	0.0003	0.0044	0.0050
RMSE	0.0003	0.0044	0.0050
Relative RMSE	2.38	29.55	**0.57**

*Note*

The bold values emphasize the negative value and the smallest relative RMSE value for the difference of the estimated CDD values.

Signif. code: *** 0.005 ** 0.01 * 0.05 ·  0.1.

### 1.9 fMRI data and methods

We obtained fMRI data from the OpenfMRI database (Poldrack et al., 2013) (http://www.openfmri.org). The accession number of the data is ds000228, and the data are available at https://openfmri.org/dataset/ds000228/. They consist of anatomical and functional MRI data of 3‐ to 12‐year‐old children and adults during the viewing of a short animated film.

### 1.10 Participants

The original data set consists of 155 participants, including children who are 3‐12 years old and adults who are 18–39 years old. This type of data is precious because it includes participants of a wide range of ages. Among all of the participants, we had to remove 26 participants during the preprocessing step (see Section *Preprocessing of fMRI Data*), so we used the data of the remaining 129 participants. In this study, we classified the participants by three age groups (3–5 year‐old, 7–12 year‐old, adult) and two gender groups (F, M). Table 3 shows the number of participants in each subgroup by a 3 × 2 classification table.

**Table 3:** The 3 ×2 classification of the participants by age and gender

		Gender		Total
		F	M	
	3–5 year‐old	34	27	61
Age	7–12 year‐old	18	20	38
	Adult	19	11	30
	Total	71	58	129

### 1.11 Experimental paradigm

The participants of the study watched a silent version of Disney Pixar's “Partly Cloudy,” a 5.6‐min animated movie. The movie was preceded by 10 s of rest, and the participants were instructed to remain still while watching the movie. The MRI data were acquired by using a 3‐Tesla Siemens TIM Trio scanner. For each participant, a total of 168 scans of whole‐brain images were acquired (repetition time (TR) = 2,000 ms, echo time (TE) = 30 msec, number of slices = 32, slice thickness = 3.3 mm, matrix size = 64 × 64, voxel dimension = 3 mm × 3 mm ×  3.3 mm).

### 1.12 Preprocessing of fMRI data

The functional MRI data were preprocessed by using the R package spm12r (Muschelli, 2018), which provides wrapper functions for Statistical Parametric Mapping (SPM) version 12 from the Wellcome Trust Centre for Neuroimaging (Ashburner et al., 2017). First, all volumes of fMRI data were spatially realigned to the average volume. Slice‐timing correction was performed after the realignment step. The fMRI was then spatially normalized to the MNI template, which was carried out by indirect steps consisting of (a) realignment of the anatomical MR images (aMRI) along the anterior commissure (AC) posterior commissure (PC) line, (b) co‐registration of aMRI to the mean fMRI image, and (c) segmentation of co‐registered aMRI into six different regions, which produced a transformation for the spatial normalization of fMRI images.

### 1.13 Selection of brain regions

We selected seven voxel locations on the cortex in the left hemisphere and another seven voxel locations symmetrically in the right hemisphere and named them based on Brodmann areas (Brodmann, 1909, 2006). The fourteen selected locations are denoted by seven capital letters (A, B, C, D, E, F, and G) and the prefixes “L” or “R” (which denotes the left or right) as described in Table 4. The exact coordinates of the selected brain locations are also listed in Table 4, which have been defined according to the Brodmann areas in the MNI template by Lacadie, Fulbright, Constable, and Papademetris (2008).

**Table 4:** The node symbols, Brodmann area (BA) numbers, abbreviated names, descriptions of the selected seven regions, and $\left(x,y,z\right)$‐coordinates under the MNI template in the brain cortex. The node symbols of each region correspond to the order of the nodes in connectivity networks in this paper. The two $\left(x,y,z\right)$‐coordinates represent the locations in the left and right hemispheres, respectively

Node	BA No.	Name	Description	MNI‐$\left(x,y,z\right)$
A	BA 04	M1	Primary Motor Cortex	$\left(-36,-17,44\right),\left(38,-18,45\right)$
B	BA 08	FEF	Frontal Eye Fields	$\left(-23,24,44\right),\left(22,26,45\right)$
C	BA 10	aPFC	Anterior Prefrontal Cortex	$\left(-23,55,4\right),\left(23,55,7\right)$
D	BA 18	V2	Visual Association Area	$\left(-19,-92,2\right),\left(29,-92,2\right)$
E	BA 20	ITG	Inferior Temporal Gyrus	$\left(-47,-14,-34\right),\left(48,-17,-31\right)$
F	BA 23	vPCC	Ventral Posterior Cingulate Cortex	$\left(-10,-45,24\right),\left(9,-45,24\right)$
G	BA 38	TP	Temporopolar Area	$\left(-43,13,-30\right),\left(40,11,-30\right)$

**Figure 5:**
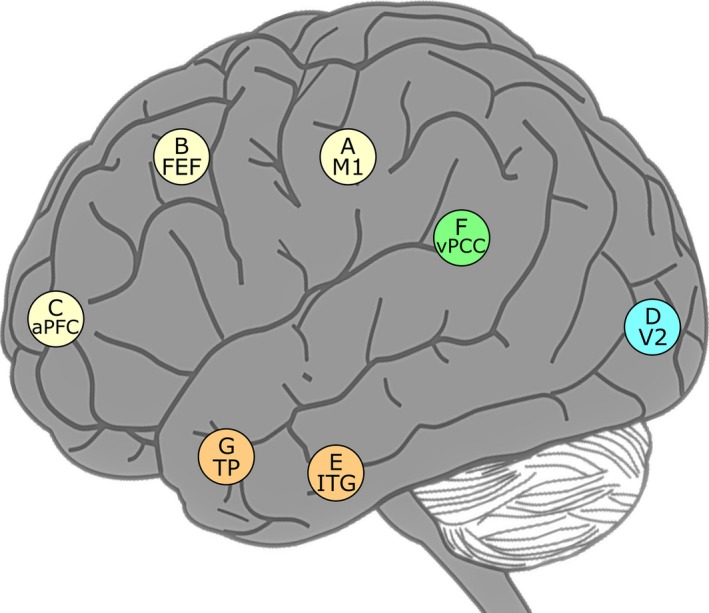
Each location of the selected seven voxels on the cortex is indicated by a circle with a label and color and illustrated in the lateral view. The selected voxels belong to a certain area among the Brodmann areas of the brain. The color of each circle in the figure indicates that it belongs to either the frontal lobe (yellow), temporal lobe (orange), occipital lobe (cyan), or limbic lobe (green)

For the selection of the brain regions, we considered the stimulus type, previous studies, and spatial distances between the regions. Since movie watching gives visual stimulus, we selected the frontal eye fields (FEF) and the visual association area (V2). In regard to the visual stimulus, identifying the connectivity of the primary motor cortex (M1) with other areas can be of potential importance in clinical neurophysiology, for example, visuomotor network (Archer et al., 2018). Richardson, Lisandrelli, Riobueno‐Naylor, and Saxe (2018) identified brain regions related to thinking about pain and the minds of others by using the same fMRI data sets, which included the posterior cingulate cortex (PCC) and prefrontal cortex (PFC). Other selected areas were included in the analysis by considering their spatial distribution. The selected areas are not spatially adjacent, and they are distributed over the brain cortex as depicted in Figure 5.

Note that during the preprocessing steps based on the SPM, we tried to avoid spatial smoothing of the fMRI. Instead, for each selected voxel location, we took a spatial average of the fMRI data values in the 5 × 5 × 5 cubic region centered at the selected voxel location. We have checked that the fourteen cubic regions in both hemispheres do not overlap.

In addition, we removed the fMRI data of 26 participants because some of the fourteen brain locations were not available in their original raw fMRI images. We suspect that an automatic process of removing face and neck areas by the OpenfMRI project has removed larger areas in the images than expected.

Moreover, we removed the first ten seconds (i.e., five scans) from the fMRI data of each participant, in order to stabilize signals and remove the resting period before the movie‐watching session started.

### 1.14 Statistical analysis

#### 1.14.1 Connectivity indices and the multiple hypothesis test

For the analysis of group differences, we estimated three connectivity statistics based on the CDDs between each pair of selected brain regions. That is, for an ordered pair $\left(U,V\right)$ of brain regions, we can estimate the CDDs from *U* to *V* ($\rho_{U\to V}^{2}$), from *V* to *U* ($\rho_{V\to U}^{2}$), and their difference ($\Delta \rho_{U,V}^{2}=\rho_{U\to V}^{2}-\rho_{V\to U}^{2}$).

In addition, we constructed a network of the selected brain regions based on the estimated CDD measures for each participant. The network structure was determined as a directed network allowing for bidirectional connections. A directed connection, from a brain region *U* to another region *V*, was pruned if the absolute value of the CDD measure was less than a threshold determined adaptively by the false discovery rate (FDR) procedure of Strimmer (2008) in order to remove spurious connections. The FDR procedure conducts a multiple hypothesis test, where we consider a set of null hypotheses that the true CDD values are zero, that is, $\rho_{U\to V}^{2}=0$ for all pairs *U* ≠ *V* of the fourteen brain regions. First, the estimated CDDs are transformed to an approximate normal distribution via Fisher's *z*‐transformation as $z=\mathrm{atanh}\left(\sqrt{\rho^{2}}\right)$. Second, the distribution of the (transformed) CDDs is approximately represented as a mixture distribution

$$f\left(z\right)=\eta f_{0}\left(z;\sigma \right)+\left(1-\eta \right)f_{A}\left(z\right),\;0\leq \eta \leq 1,$$

where *f*_0_ is the density function of a null distribution and *f*_A_ is an alternative distribution. The null distribution, $f_{0}\left(\cdot ;\sigma \right)$, is the normal density function with a mean of zero and standard deviation of *σ*, which represents the distribution of the estimated CDDs when the true CDD value is zero. The shape parameter *σ* > 0 and the portion parameter *η* are automatically estimated based on the given (transformed) CDDs. Finally, the local FDR score is computed by

$$\mathrm{fdr}\left(\rho \right)=\mathrm{Pr}\left(\text{true CDD is zero}\;|z\right)=\frac{\hat{\eta }f_{0}\left(z;\hat{\kappa }\right)}{f\left(z\right)}.$$

Typically, a directed connection from *U* to *V* is removed if $\mathrm{fdr}\left(\rho_{U\to V}\right)\geq 0.2.$

**Figure 6:**
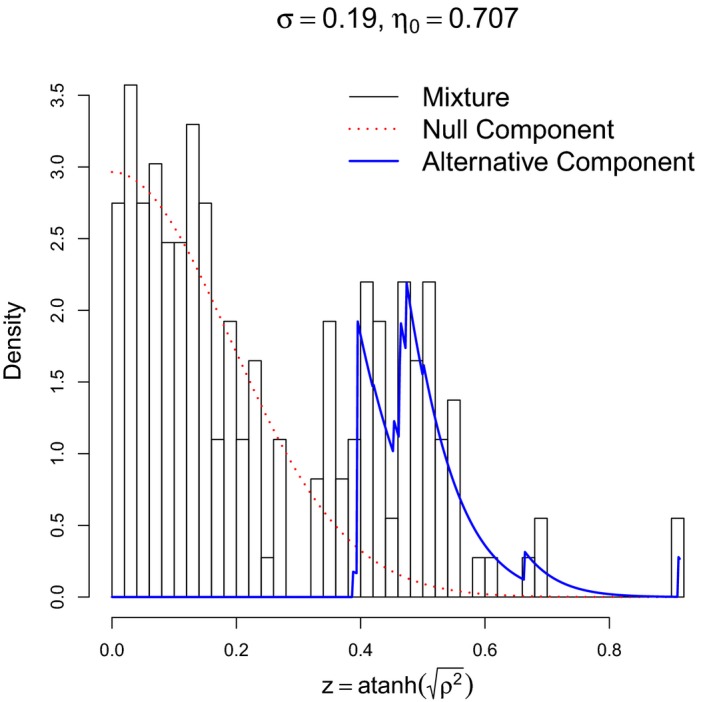
An empirical distribution of estimated CDDs for a participant selected from the group of Adult and F (black). The CDDs have been transformed to normal distributions via Fisher's z‐transformation. The null and alternative distributions estimated by the FDR procedure of Strimmer (2008) are shown by the red dotted curve and blue straight line, respectively. On the top of the figure, the standard deviation (*σ* = 0.19) and proportion (*η* = 0.707) of the null distribution are displayed

Figure 6 illustrates an example of the mixture distribution obtained for a participant belonging to the Adult‐Female group. The null and alternative distributions are depicted by the red dotted line and the blue straight line, respectively.

#### 1.14.2 Nonparametric tests of group differences

We applied nonparametric tests for group differences based on a measure of network structure and a connectivity measure. As a measure of network structure, we used the total‐degree, in‐degree, and out‐degree of each node, which are a kind of centrality measure in graph theory. As a measure of connectivity, we used three estimates based on the CDDs between each pair of brain regions $\left(U,V\right)$, that is, $\rho_{U\to V}^{2}$, $\rho_{V\to U}^{2}$, and $\rho_{U\to V}^{2}-\rho_{V\to U}^{2}$. Since the distributions of the degrees and the CDDs are skewed and not normally distributed, we applied nonparametric analysis of variance (ANOVA) methods for group analysis. Specifically,

1. Kruskal–Wallis (KW) rank‐sum test was applied for each factor. The software is available in the R package stats as the function kruskal.test().
2. Quantile ANOVA, which refers to one‐way ANOVA based on quantiles, was applied for each factor (Wilcox, 2012). It is known to work well when there are tie values in the data, and it is implemented in the R package WRS2 as the function Qanova() (Mair & Wilcox, 2017).
3. Robust two‐way ANOVA based on medians was applied for the factors with interaction effects (Wilcox, 2012). It is implemented in the R package WRS2 as the function med2way() (Mair & Wilcox, 2017).

There can be other methods for a nonparametric test of group differences; see, for example, Feys (2016) for nonparametric ANOVA using R software. We remark that there are many tie values in our data, for example, the node degrees, which prohibit some nonparametric test methods from being adopted.

## 2 RESULTS

### 2.1 Subject‐level analysis

**Figure 7:**
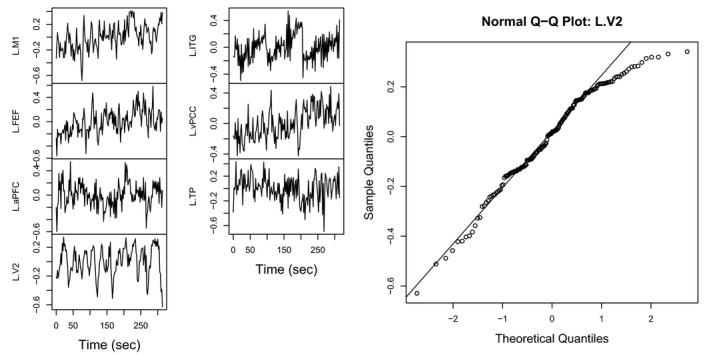
A preprocessed sample fMRI time series for a participant selected from the group of Adult and F (left panel), and a normal Q‐Q plot for a sample fMRI time series from the BA18 V2 in the left hemisphere (right panel)

We selected a sample participant from the group of Adult and F (female). The time series data from the seven predefined regions in the left hemisphere of the selected participant are shown in the left panel of Figure 7. The time series are not weakly stationary, that is, the mean and the variance are not constant over time. The normal Q‐Q plot for the fourth time series which corresponds to the BA18 L.V2 region (“L” or “R” means the left or right hemisphere) is shown in the right panel of Figure 7. The time series is not normally distributed as there are points with relatively small quantile values. The Shapiro–Wilks test of normality for this time series reported a test statistic of *W* = 0.9680 and a *p*‐value of 0.0010, which implies that the data are not normally distributed. Note that weak stationarity and normality are typical assumptions of a wide range of dynamical system models.

**Figure 8:**
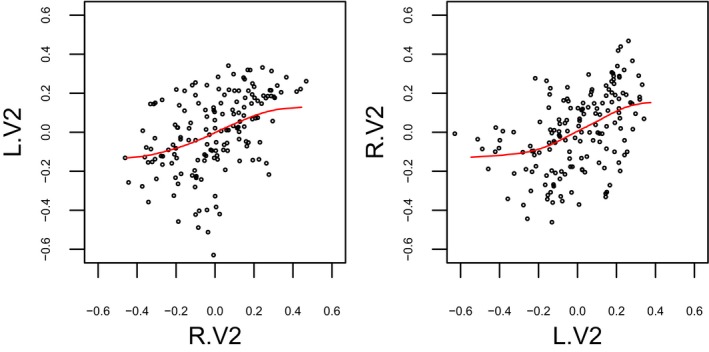
Sample scatter plots for the fMRI time series from the regions BA18 V2 in both hemispheres for a participant selected from the group of Adult and F (female). The red line in each plot represents the regression line by beta regression

Scatter plots for the time series of a pair of two selected regions BA18 L.V2 and BA18 R.V2 are shown in Figure 8. The red line in each panel represents a regression line determined by beta regression. Note that the beta regression lines are nonlinear curves parameterized by Equation 3.

Table 5 shows the estimated CDDs for selected pairs of the fourteen brain regions having local FDR scores <0.2. Supporting Information Table S1 contains the estimated CDDs for all $\left(13\times 14\right)/2=91$ pairs. For each pair $\left(U,V\right)$, we computed CDDs $\rho_{U\to V}^{2}$, $\rho_{V\to U}^{2}$, and their difference $\Delta \rho^{2}=\rho_{U\to V}^{2}-\rho_{V\to U}^{2}$. The sign of the difference determines which of the copula regressions *U* → *V* and *V* → *U* have higher prediction power. In the table, we have switched the order of *U* and *V* so that the regression *U* → *V* has a higher prediction power.

**Table 5:** Estimates of the copula directional dependences for a participant selected from the group of Adult and F (female). Δρ^2^ denotes the difference $\Delta \rho^{2}=\rho_{U\to V}^{2}-\rho_{V\to U}^{2}$. $\mathrm{LB}\left(\Delta \rho^{2}\right)$ and $\mathrm{UB}\left(\Delta \rho^{2}\right)$ represent the lower bound and the upper bound of the 95% confidence interval for the difference, Δρ^2^, respectively. The table contains a list of selected pairs of the brain regions having local FDR scores <0.2. The lower bounds, $\mathrm{LB}\left(\Delta \rho^{2}\right)$, larger than zero are written in bold font

Pair No.	Brain region *U*	Brain region *V*	$\rho_{U\to V}^{2}$	$\rho_{V\to U}^{2}$	$\Delta \rho_{U,V}^{2}$	$LB\left(\Delta \rho^{2}\right)$	$UB\left(\Delta \rho^{2}\right)$
13	R.A M1	L.A M1	0.214	0.202	0.012	**0.007**	0.013
14	R.C aPFC	R.B FEF	0.166	0.158	0.008	**0.005**	0.011
15	R.B FEF	R.D V2	0.186	0.153	0.033	**0.031**	0.037
17	R.F vPCC	R.B FEF	0.226	0.217	0.009	**0.007**	0.013
18	R.G TP	R.B FEF	0.194	0.181	0.014	**0.012**	0.017
24	L.B FEF	R.B FEF	0.359	0.296	0.063	**0.056**	0.062
25	R.B FEF	L.A M1	0.152	0.141	0.011	**0.008**	0.012
27	R.C aPFC	R.E ITG	0.149	0.141	0.009	**0.004**	0.010
28	R.F vPCC	R.C aPFC	0.255	0.245	0.010	**0.004**	0.014
29	R.G TP	R.C aPFC	0.351	0.337	0.014	**0.007**	0.015
31	L.F vPCC	R.C aPFC	0.227	0.207	0.020	**0.012**	0.020
34	L.C aPFC	R.C aPFC	0.245	0.220	0.024	**0.021**	0.026
35	R.C aPFC	L.B FEF	0.222	0.188	0.034	**0.030**	0.036
36	R.C aPFC	L.A M1	0.285	0.245	0.040	**0.032**	0.040
38	R.D V2	R.F vPCC	0.235	0.234	0.001	−0.001	0.006
39	R.G TP	R.D V2	0.240	0.200	0.040	**0.038**	0.046
41	L.F vPCC	R.D V2	0.170	0.144	0.026	**0.021**	0.028
43	L.D V2	R.D V2	0.237	0.206	0.032	**0.027**	0.034
45	L.B FEF	R.D V2	0.166	0.145	0.021	**0.017**	0.023
46	R.D V2	L.A M1	0.195	0.188	0.007	**0.006**	0.013
48	R.G TP	R.E ITG	0.251	0.211	0.040	**0.035**	0.041
49	R.E ITG	L.G TP	0.161	0.149	0.012	**0.007**	0.013
56	R.G TP	R.F vPCC	0.217	0.194	0.022	**0.020**	0.027
58	R.F vPCC	L.F vPCC	0.522	0.520	0.002	−0.002	0.008
62	R.F vPCC	L.B FEF	0.180	0.166	0.014	**0.010**	0.016
63	R.F vPCC	L.A M1	0.216	0.203	0.013	**0.007**	0.014
65	R.G TP	L.F vPCC	0.163	0.146	0.017	**0.014**	0.021
69	L.B FEF	R.G TP	0.157	0.157	0.000	−0.001	0.006
70	L.A M1	R.G TP	0.198	0.188	0.009	**0.008**	0.014

In addition, in order to provide statistical significance of the determined regression direction, that is, *U* → *V*, bootstrap resampling was applied to yield a 95% confidence interval (CI) for the difference $\Delta \rho_{U\to V}^{2}$. In the table, since the order of *U* and *V* has changed to have Δ*ρ* ^2^ > 0, we only need to check whether the lower bound of the 95% CI is positive or not. In Table 5, which shows a list of the selected pairs with $\mathrm{fdr}\left(\rho \right)<0.2$, there are only three regression directions which are not statistically significant, that is, the directions #38 R.D V2 → R.F vPCC, #58 R.F vPCC → L.F vPCC, and #69 L.B FEF → R.G TP.

**Figure 9:**
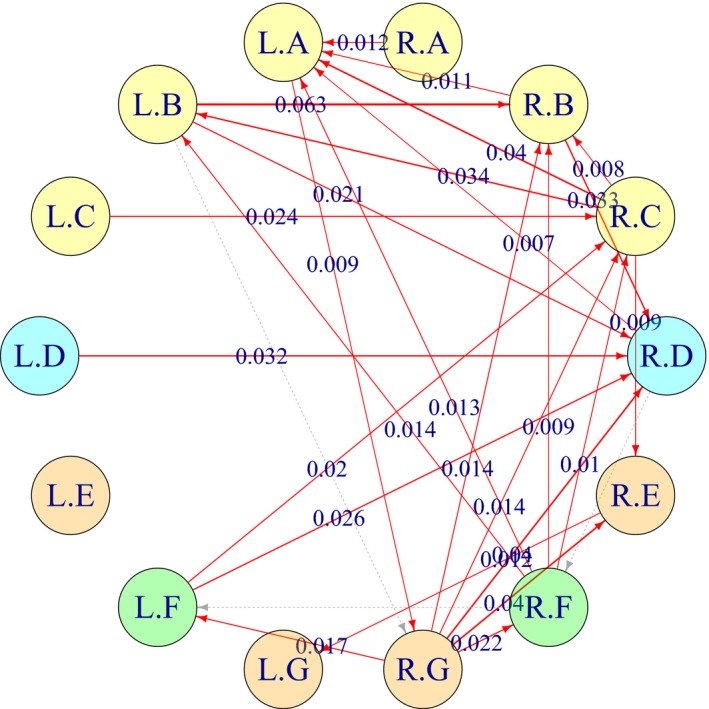
A sample connectivity network of brain regions for a participant selected from the group of Adult and F (female). Each of the edges in the directed network was pruned if the local FDR score was greater than or equal to 0.2, that is, $\mathrm{fdr}\left(\rho_{U\to V}\right)\geq 0.2$. The “R” and “L” in the node labels represent the right and left hemispheres, and the letters from “A” to “G” represent the brain regions described in Table 4. An arrow from brain regions *U* to *V* represents the difference of the estimated CDDs, $\Delta \rho_{U,V}^{2}$, for $\rho_{U\to V}^{2}>\rho_{V\to U}^{2}$, and it is colored in red if $\Delta \rho_{U,V}^{2}$ was significantly larger than zero, that is, $LB\left(\Delta \rho^{2}\right)>0$

A directed network of the fourteen brain regions inferred based on the estimated CDDs in Table 5 is illustrated in Figure 9. The edges in the directed network were pruned based on the local FDR score, that is, an arrow from a node *U* to *V* indicates that the local FDR score $\mathrm{fdr}\left(\rho_{U\to V}\right)$ is <0.2. As a result, the obtained directed network is bidirectional, which allows us to compare the relative strengths of the directional dependences. For simplicity, between the two opposite directions *U* → *V* and *V* → *U*, the figure shows only the direction *U* → *V* having the larger CDD value, that is, $\rho_{U\to V}^{2}>\rho_{V\to U}^{2}$. An arrow is colored in red if the difference of the CDD values $\Delta \rho_{U,V}^{2}$ is significantly larger than zero, that is, $LB\left(\Delta \rho^{2}\right)>0$.

**Figure 10:**
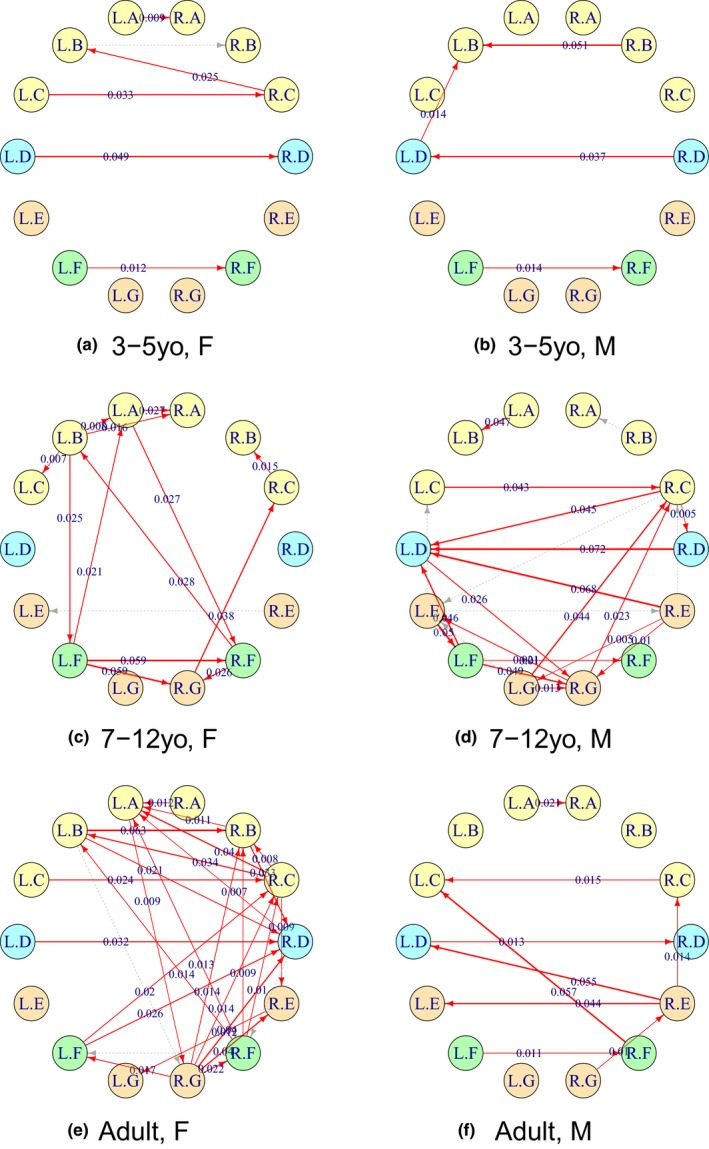
Connectivity networks of brain regions for participants selected from each of the six groups classified by three age levels and two gender levels. The connectivity networks for every participant are available at http://github.com/namgillee/ CDDforFMRI. The “R” and “L” in the node labels represent the right and left hemispheres, and the letters from “A” to “G” represent the brain regions described in Table 4. An arrow from brain regions *U* to *V* represents the difference of the estimated CDDs, $\Delta \rho_{U,V}^{2}$, for $\rho_{U\to V}^{2}>\rho_{V\to U}^{2}$, and it is colored in red if $\Delta \rho_{U,V}^{2}$ was significantly larger than zero, that is, $LB\left(\Delta \rho^{2}\right)>0$

Figure 10 illustrates the CDD connectivity networks obtained for six participants selected from each of the Age and Gender subgroups. The connectivity networks for every participant are available at https://github.com/namgillee/CDDforFMRI.

### 2.2 Group‐level analysis

In order to verify the validity of the suggested approach in the inference of brain connectivity, we considered all of the 129 participants whose fourteen selected brain regions had no missing data in fMRI images. The participants were grouped into three age groups (3–5 year‐old, 7–12 year‐old, Adult) and two gender groups (F, M); see Table 3. The chi‐square test of independence of the two factors yielded *χ* ^2^ statistics of 1.7496 with *df* = 2 and a *p*‐value of 0.417, which implies that the two factors are statistically independent.

### 2.3 Group analysis using node degree

Based on the connectivity network structures inferred by local FDR scores of CDD measures, the node degree corresponding to each brain region was analyzed by nonparametric ANOVA tests of group differences. Among the graph theoretic measures, node degree is the simplest and most basic centrality measure.

**Figure 11:**
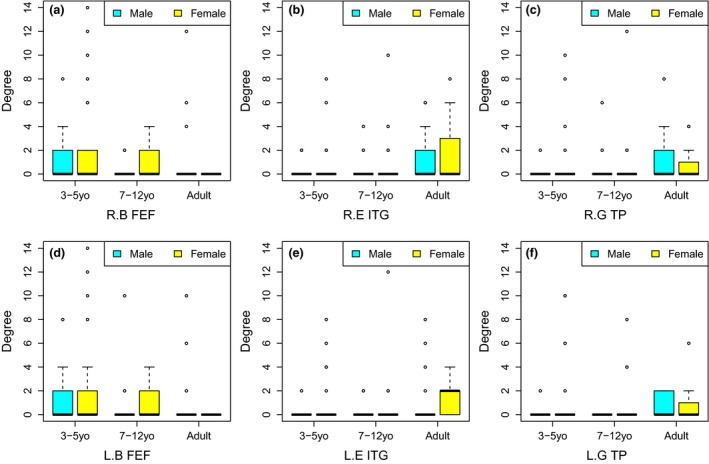
Boxplots for comparing distributions of total‐degree between groups

Table 6 shows the results of nonparametric ANOVA tests using total‐degrees. The nonparametric tests are one‐way tests such as Kruskal–Wallis and Quantile ANOVA. Instead of applying a multiple comparisons correction (MCC) to select significantly small *p*‐values, all *p*‐values are shown in Table 6. We can see that the total‐degree of FEF, ITG, and TP in both hemispheres is significantly affected by age. The boxplots of the total‐degree of the six brain regions over different factor groups are shown in Figure 11. In the figure, we can clearly see the effect of age.

**Table 6:** *p*‐values by nonparametric ANOVA tests using total‐degree

Name	Kruskal–Wallis		Quantile–ANOVA		Median	
	Age	Gender	Age	Gender	Age	Gender
R.A M1	0.986	0.407	0.765	0.613	1.000	1.000
R.B FEF	**0.036** *	0.301	0.705	0.568	1.000	1.000
R.C aPFC	0.489	0.053·	0.612	0.462	1.000	1.000
R.D V2	0.201	0.578	0.713	0.667	1.000	1.000
R.E ITG	**0.019** *	0.482	0.523	0.627	1.000	1.000
R.F vPCC	0.552	0.136	0.870	0.493	1.000	1.000
R.G TP	**0.022** *	0.463	0.470	0.550	1.000	1.000
L.A M1	0.542	0.204	0.685	0.498	1.000	1.000
L.B FEF	**0.044** *	0.192	0.638	0.520	1.000	1.000
L.C aPFC	0.524	0.137	0.627	0.485	1.000	1.000
L.D V2	0.275	0.577	0.732	0.788	1.000	1.000
L.E ITG	**0.048** *	**0.017** *	0.655	0.420	1.000	**0.021** *
L.F vPCC	0.590	0.116	0.790	0.487	1.000	1.000
L.G TP	**0.031** *	0.090·	0.647	0.452	1.000	1.000

*Note*

Bold values emphasize the statistically significant *p*‐values (<0.05).

Signif. code: ***0.005 **0.01 *0.05 ·  0.1.

### 2.4 Group analysis using connection strengths

The CDD from one brain region to another measures the connection strength in the brain network. We applied nonparametric ANOVA tests using the CDD measures $\rho_{U\to V}^{2}$, $\rho_{V\to U}^{2}$, and the difference $\Delta \rho_{U,V}^{2}=\rho_{U\to V}^{2}-\rho_{V\to U}^{2}$, where $\left(U,V\right)$ represents a pair of brain regions. We suppose that the brain regions are ordered by the node numbers in Table 4.

Table 7 summarizes the results of the nonparametric ANOVA tests using the CDD measures from a brain region *U* to another region *V* with *U* < *V*. In this section, we only present the results for the CDD measures having *p*‐values smaller than 0.05 due to the space limit. All the results for the CDDs from *U* to *V* with *U* < *V* are available at https://github.com/namgillee/CDDforFMRI.

**Table 7:** *p*‐values for $\rho_{U\to V}^{2}$ with U < *V*

Connection	Kruskal–Wallis		Quantile–ANOVA		Median		
	Age	Gender	Age	Gender	Age	Gender	Age:Gender
R.M1 → L.aPFC	0.421	0.078 ·	0.525	0.008**	0.903	0.073	0.247
R.M1 → L.FEF	0.027*	0.405	0.068 ·	0.643	**0.009** **	0.902	0.072 ·
R.FEF→ R.vPCC	0.985	0.580	0.988	0.780	0.828	0.900	**0.000** ***
R.FEF→ L.FEF	**0.005** ***	0.176	**0.005** **	0.425	0.017*	0.755	0.425
R.FEF→ L.M1	**0.005** ***	0.551	0.020*	0.507	0.052 ·	0.258	0.189
R.aPFC→ R.TP	**0.006** **	0.925	**0.008** **	0.943	0.064 ·	0.653	0.016*
R.ITG→ R.TP	**0.000** ***	0.906	**0.000** ***	0.582	**0.007** **	0.874	0.250
R.vPCC→ L.FEF	0.297	0.366	0.378	0.662	0.089 ·	0.028*	**0.000** ***
L.vPCC→ L.FEF	0.381	0.106	0.098 ·	0.233	0.066 ·	0.081 ·	**0.005** **

*Note*

Bold values emphasize the statistically significant *p*‐values (<0.01).

Signif. code: ***0.005 **0.01 *0.05 · 0.1.

**Figure 12:**
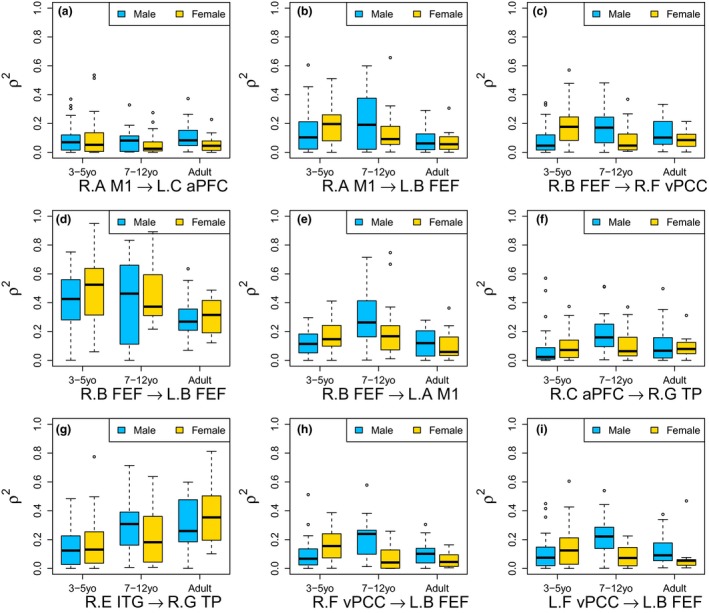
Boxplots for comparing distributions of $\rho_{U\to V}^{2}$ with *U* < *V* between groups

In Table 7, the nine CDD connection strengths are highly significantly affected by the group differences. First, the CDD from R.M1 to L.FEF and the CDD from R.FEF to L.FEF are significantly decreasing as the age of the participant group increases; see Figure 12b,d for boxplots to clearly compare the distributions between different age groups and gender groups. The CDD from R.FEF to L.M1 and the CDD from R.FEF to R.TP are significantly high for the age group of 7–12 year‐old; see Figure 12e,f. The CDD from R.ITG to R.TP is significantly increasing as the age of the participant group increases; see Figure 12g. Second, the CDDs from R.FEF to R.vPCC, from R.vPCC to L.FEF, and from L.vPCC to L.FEF are significantly affected by the interaction effect of age and gender factors; see Figure 12c,h,i for boxplots of the CDD values between the different groups. Next, the CDD from R.M1 to L.aPFC shows significantly distinctive distributions between the gender groups; see Figure 8a for the boxplot to see the distribution change.

In Supporting Information Tables S2 and S3 and Figures S1 and S2, we also provide the results of the nonparametric ANOVA tests using $\rho_{V\to U}^{2}$ and $\Delta \rho_{U,V}^{2}$ for *U* < *V*, where we present the results for *p*‐values smaller than 0.05 due to the space limit. All the results are available at https://github.com/namgillee/CDDforFMRI. The results using $\rho_{V\to U}^{2}$ with *U* < *V* in Supporting Information Table S2 are consistent with the results presented in this section.

## 3 DISCUSSION AND CONCLUSIONS

In this paper, we proposed a new method for discovering directed connectivity between whole‐brain regions using fMRI data, which is called copula directional dependence (CDD). The proposed method is based on the copula regression model using beta regression, which can effectively and flexibly detect nonlinear relationships between brain regions without strict assumptions on specific distributions.

Compared to dynamical system modeling approaches for discovering effective connectivity, the CDD considers realistic cases where neuroimaging techniques such as fMRI have much lower sampling frequencies compared to the speed of the underlying neurophysiological signal transfer, so that it may be difficult to identify the true causal relationships in an fMRI study. Instead, the CDD infers networks with bidirectional connectivity, so we can compare the relative strengths of the directional dependences and provide statistical significance of the directions between each pair of brain regions. In this paper, we explained a practical process of using the ordinary nonparametric bootstrap for determining the significance of the directional dependences. The CDD can be interpreted as a directional dependence relationship or a predictive power, which can be further interpreted as a causal relationship determined based on regression models using observational data.

The proposed method can be used for exploratory data analysis where specific task‐related brain regions and functions are previously unknown, but the goal can be a search for “biomarkers” related to specific tasks or experimental conditions. We applied the CDD method to fMRI data of 129 participants watching a Pixar silent animated movie. A noticeable characteristic of this data is that it includes participants of a wide range of ages, from 3 years old to 39 years old. We computed the CDD measures between every ordered pair of brain regions using preprocessed fMRI data and yielded bidirectional connectivity networks. Based on the computed CDD measures and the connectivity networks, we could conduct a set of nonparametric ANOVA tests for group differences and identify specific brain regions and connection strengths which are highly significantly affected by physiological conditions such as age, gender, and their interaction effect.

Specifically, based on the results of the fMRI data analyses for group differences presented in Section *Group‐Level Analysis*, we could identify three subnetworks of brain regions, each of which consists of the brain regions (nodes) and directed connections (edges) which are highly significantly affected by age, gender, or their interaction effect as follows:

1 The *age‐sensitive network* has the directed connections whose strength changes over different age groups:
  - The CDDs of the connections R.M1 → L.FEF and R.FEF → L.FEF are decreased for older age groups.
  - The CDD of the connection R.ITG → R.TP is increased for older age groups.
2 In addition, the node degrees of FEF, ITG, and TP in the left and right hemispheres are highly significantly affected by age.
3 The *gender‐sensitive network* has two brain regions, R.M1 and L.aPFC, with the directed connection R.M1 → L.aPFC. Its connection strength is higher for the male subgroups than for the female subgroups.
4 The *interaction effect‐sensitive network* has four brain regions, R.FEF, L.FEF, R.vPCC, and L.vPCC, with the directed connections R.FEF→R.vPCC,R.vPCC→L.FEF, and L.vPCC → L.FEF.

Since the suggested CDD measure can be computed and applied to any other experimental paradigms, task conditions, and numbers of brain regions, it can be widely used in future works and in clinical trials.
