# Supplementary material for: Copula directional dependence for inference and statistical analysis of whole‐brain connectivity from fMRI data
Source: Brain Behav. 2018 Dec 27;9(1):e01191. doi: 10.1002/brb3.1191 (PMC6346668; doi:10.1002/brb3.1191)

Supplementary Materials for

Copula directional dependence for inference and statistical analysis of whole brain connectivity from fMRI data

Namgil Lee, Jong-Min Kim[1]

Tables and Figures

Table S1. Estimates of the copula directional dependence (CDD) for a participant selected from the group of Adult and F (female). $\Delta\rho^2$ *denotes the difference* $\Delta\rho^2 = \rho^2_{U\to V} - \rho^2_{V\to U}$. $LB(\Delta\rho^2)$ *and* $UB(\Delta\rho^2)$ *represent the lower bound and the upper bound of the 95% confidence interval for the difference,* $\Delta\rho^2$*, respectively. A pair of brain regions,* (U, V)*, is written in bold font if either* $\rho^2_{U\to V}$ *or* $\rho^2_{V\to U}$ *has a local FDR score less than 0.2.*

Edge No	Brain region U	Brain region V	$\rho^2_{U\to V}$	$\rho^2_{V\to U}$	$\Delta\rho^2_{U,V}$	$LB(\Delta\rho^2)$	$UB(\Delta\rho^2)$
1	R.B FEF	R.A M1	0.009	0.003	0.006	0.005	0.007
2	R.A M1	R.C aPFC	0.069	0.065	0.004	0.001	0.005
3	R.A M1	R.D V2	0.016	0.015	0.001	0.000	0.003
4	R.E ITG	R.A M1	0.051	0.035	0.017	0.015	0.019
5	R.A M1	R.F vPCC	0.099	0.099	0.000	-0.002	0.003
6	R.G TP	R.A M1	0.004	0.002	0.003	0.002	0.003
7	R.A M1	L.G TP	0.000	0.000	0.000	-0.001	0.000
8	R.A M1	L.F vPCC	0.056	0.048	0.007	0.004	0.008
9	L.E ITG	R.A M1	0.033	0.021	0.012	0.010	0.013
10	L.D V2	R.A M1	0.022	0.018	0.004	0.002	0.004
11	R.A M1	L.C aPFC	0.003	0.000	0.003	0.002	0.004
12	L.B FEF	R.A M1	0.004	0.001	0.003	0.003	0.004
**13**	**R.A M1**	**L.A M1**	**0.214**	**0.202**	**0.012**	**0.007**	**0.013**

[1] Corresponding author. Email: jongmink@morris.umn.edu

**14**	**R.C aPFC**	**R.B FEF**	**0.166**	**0.158**	**0.008**	**0.005**	**0.011**
**15**	**R.B FEF**	**R.D V2**	**0.186**	**0.153**	**0.033**	**0.031**	**0.037**
16	R.B FEF	R.E ITG	0.015	0.012	0.003	0.001	0.003
**17**	**R.F vPCC**	**R.B FEF**	**0.226**	**0.217**	**0.009**	**0.007**	**0.013**
**18**	**R.G TP**	**R.B FEF**	**0.194**	**0.181**	**0.014**	**0.012**	**0.017**
19	R.B FEF	L.G TP	0.034	0.022	0.011	0.011	0.014
20	L.F vPCC	R.B FEF	0.112	0.112	0.000	-0.003	0.002
21	R.B FEF	L.E ITG	0.003	0.001	0.002	0.002	0.004
22	L.D V2	R.B FEF	0.017	0.010	0.008	0.007	0.009
23	R.B FEF	L.C aPFC	0.001	0.001	0.000	0.000	0.001
**24**	**L.B FEF**	**R.B FEF**	**0.359**	**0.296**	**0.063**	**0.056**	**0.062**
**25**	**R.B FEF**	**L.A M1**	**0.152**	**0.141**	**0.011**	**0.008**	**0.012**
26	R.D V2	R.C aPFC	0.136	0.120	0.016	0.011	0.017
**27**	**R.C aPFC**	**R.E ITG**	**0.149**	**0.141**	**0.009**	**0.004**	**0.010**
**28**	**R.F vPCC**	**R.C aPFC**	**0.255**	**0.245**	**0.010**	**0.004**	**0.014**
**29**	**R.G TP**	**R.C aPFC**	**0.351**	**0.337**	**0.014**	**0.007**	**0.015**
30	L.G TP	R.C aPFC	0.045	0.042	0.003	0.001	0.004
**31**	**L.F vPCC**	**R.C aPFC**	**0.227**	**0.207**	**0.020**	**0.012**	**0.020**
32	L.E ITG	R.C aPFC	0.002	0.001	0.002	0.000	0.002
33	L.D V2	R.C aPFC	0.017	0.015	0.002	0.002	0.004
**34**	**L.C aPFC**	**R.C aPFC**	**0.245**	**0.220**	**0.024**	**0.021**	**0.026**
**35**	**R.C aPFC**	**L.B FEF**	**0.222**	**0.188**	**0.034**	**0.030**	**0.036**
**36**	**R.C aPFC**	**L.A M1**	**0.285**	**0.245**	**0.040**	**0.032**	**0.040**
37	R.E ITG	R.D V2	0.005	0.005	0.000	-0.001	0.000
**38**	**R.D V2**	**R.F vPCC**	**0.235**	**0.234**	**0.001**	**-0.001**	**0.006**
**39**	**R.G TP**	**R.D V2**	**0.240**	**0.200**	**0.040**	**0.038**	**0.046**
40	R.D V2	L.G TP	0.052	0.035	0.017	0.016	0.019
**41**	**L.F vPCC**	**R.D V2**	**0.170**	**0.144**	**0.026**	**0.021**	**0.028**
42	L.E ITG	R.D V2	0.014	0.013	0.001	-0.001	0.001
**43**	**L.D V2**	**R.D V2**	**0.237**	**0.206**	**0.032**	**0.027**	**0.034**
44	L.C aPFC	R.D V2	0.002	0.000	0.002	0.001	0.002
**45**	**L.B FEF**	**R.D V2**	**0.166**	**0.145**	**0.021**	**0.017**	**0.023**
**46**	**R.D V2**	**L.A M1**	**0.195**	**0.188**	**0.007**	**0.006**	**0.013**
47	R.E ITG	R.F vPCC	0.009	0.006	0.002	0.001	0.003
**48**	**R.G TP**	**R.E ITG**	**0.251**	**0.211**	**0.040**	**0.035**	**0.041**
**49**	**R.E ITG**	**L.G TP**	**0.161**	**0.149**	**0.012**	**0.007**	**0.013**
50	L.F vPCC	R.E ITG	0.009	0.007	0.002	0.001	0.003
51	L.E ITG	R.E ITG	0.020	0.006	0.014	0.011	0.014
52	R.E ITG	L.D V2	0.003	0.001	0.002	0.000	0.002
53	R.E ITG	L.C aPFC	0.014	0.013	0.000	-0.001	0.001
54	L.B FEF	R.E ITG	0.010	0.004	0.006	0.005	0.007
55	R.E ITG	L.A M1	0.030	0.013	0.017	0.015	0.018
**56**	**R.G TP**	**R.F vPCC**	**0.217**	**0.194**	**0.022**	**0.020**	**0.027**
57	R.F vPCC	L.G TP	0.008	0.001	0.007	0.006	0.008
**58**	**R.F vPCC**	**L.F vPCC**	**0.522**	**0.520**	**0.002**	**-0.002**	**0.008**

59	L.E ITG	R.F vPCC	0.028	0.011	0.017	0.015	0.017
60	L.D V2	R.F vPCC	0.047	0.034	0.014	0.013	0.016
61	L.C aPFC	R.F vPCC	0.013	0.009	0.004	0.004	0.006
**62**	**R.F vPCC**	**L.B FEF**	**0.180**	**0.166**	**0.014**	**0.010**	**0.016**
**63**	**R.F vPCC**	**L.A M1**	**0.216**	**0.203**	**0.013**	**0.007**	**0.014**
64	R.G TP	L.G TP	0.125	0.109	0.015	0.013	0.018
**65**	**R.G TP**	**L.F vPCC**	**0.163**	**0.146**	**0.017**	**0.014**	**0.021**
66	L.E ITG	R.G TP	0.037	0.021	0.016	0.014	0.017
67	R.G TP	L.D V2	0.007	0.004	0.003	0.002	0.004
68	L.C aPFC	R.G TP	0.028	0.014	0.013	0.011	0.013
**69**	**L.B FEF**	**R.G TP**	**0.157**	**0.157**	**0.000**	**-0.001**	**0.006**
**70**	**L.A M1**	**R.G TP**	**0.198**	**0.188**	**0.009**	**0.008**	**0.014**
71	L.F vPCC	L.G TP	0.002	0.000	0.002	0.001	0.002
72	L.G TP	L.E ITG	0.049	0.032	0.018	0.016	0.020
73	L.G TP	L.D V2	0.004	0.001	0.003	0.002	0.005
74	L.G TP	L.C aPFC	0.001	0.000	0.001	0.000	0.001
75	L.G TP	L.B FEF	0.001	0.000	0.001	0.000	0.002
76	L.G TP	L.A M1	0.053	0.042	0.011	0.009	0.012
77	L.E ITG	L.F vPCC	0.015	0.014	0.001	0.001	0.003
78	L.F vPCC	L.D V2	0.070	0.069	0.001	0.000	0.004
79	L.F vPCC	L.C aPFC	0.000	0.000	0.000	0.000	0.001
80	L.B FEF	L.F vPCC	0.122	0.118	0.003	0.003	0.008
81	L.F vPCC	L.A M1	0.112	0.104	0.008	0.006	0.011
82	L.D V2	L.E ITG	0.023	0.020	0.003	0.002	0.004
83	L.E ITG	L.C aPFC	0.024	0.020	0.004	0.004	0.007
84	L.E ITG	L.B FEF	0.001	0.001	0.001	0.000	0.002
85	L.A M1	L.E ITG	0.005	0.000	0.005	0.003	0.006
86	L.C aPFC	L.D V2	0.022	0.019	0.003	0.003	0.005
87	L.D V2	L.B FEF	0.045	0.031	0.014	0.012	0.015
88	L.D V2	L.A M1	0.010	0.003	0.006	0.005	0.007
89	L.C aPFC	L.B FEF	0.015	0.005	0.010	0.009	0.011
90	L.C aPFC	L.A M1	0.003	0.002	0.000	0.000	0.001
91	L.B FEF	L.A M1	0.113	0.108	0.005	0.002	0.007

Table S2. P-values for $\rho^2_{U \leftarrow V}$ with $U < V$.

	Kruskal-Wallis		Quantile-ANOVA		Median		
Connection	Age	Gender	Age	Gender	Age	Gender	Age:Gender
R.FEF←R.vPCC	0.982	0.667	0.975	0.89	0.784	0.484	0.000 ***
R.FEF←L.FEF	0.003 ***	0.208	0.000 ***	0.225	0.022 *	0.805	0.519
R.FEF←L.M1	0.006 **	0.520	0.017 *	0.343	0.048 *	0.470	0.164
R.aPFC←R.TP	0.006 **	0.880	0.018 *	0.842	0.012 *	0.324	0.007 **
R.ITG←R.TP	0.000 ***	0.715	0.007 **	0.303	0.012 *	0.926	0.330
R.ITG←L.ITG	0.010 **	0.726	0.005 **	0.938	0.031 *	0.559	0.090 ·
R.vPCC←L.FEF	0.413	0.351	0.425	0.665	0.197	0.038 *	0.000 ***
L.vPCC←L.FEF	0.349	0.160	0.077 ·	0.170	0.041 *	0.031 *	0.005 ***
L.FEF←L.M1	0.020 *	0.363	0.017 *	0.478	0.007 **	0.597	0.104

Signif. code: *** 0.005 ** 0.01 * 0.05 · 0.1

Table S3. P-values for $\Delta\rho^2_{U,V}$.

	Kruskal-Wallis		Quantile-ANOVA		Median		
Connection (U, V)	Age	Gender	Age	Gender	Age	Gender	Age:Gender
R.M1, L.ITG	0.293	0.005 ***	0.367	0.038 *	0.737	0.214	0.522
R.FEF, L.ITG	0.983	0.010 **	0.908	0.065 *	0.840	0.559	0.719
R.ITG, L.vPCC	0.002 ***	0.224	0.027 *	0.308	0.018	0.350	0.191
R.TP, L.vPCC	0.019 *	0.413	0.007 **	0.537	0.124	0.578	0.464
L.ITG, L.FEF	0.005 **	0.332	0.060 ·	0.457	0.184	0.625	0.774

Signif. code: *** 0.005 ** 0.01 * 0.05 · 0.1

Figure S1. Boxplots for comparing distributions of $\rho^2_{V\to U}$ with $U < V$ between groups.

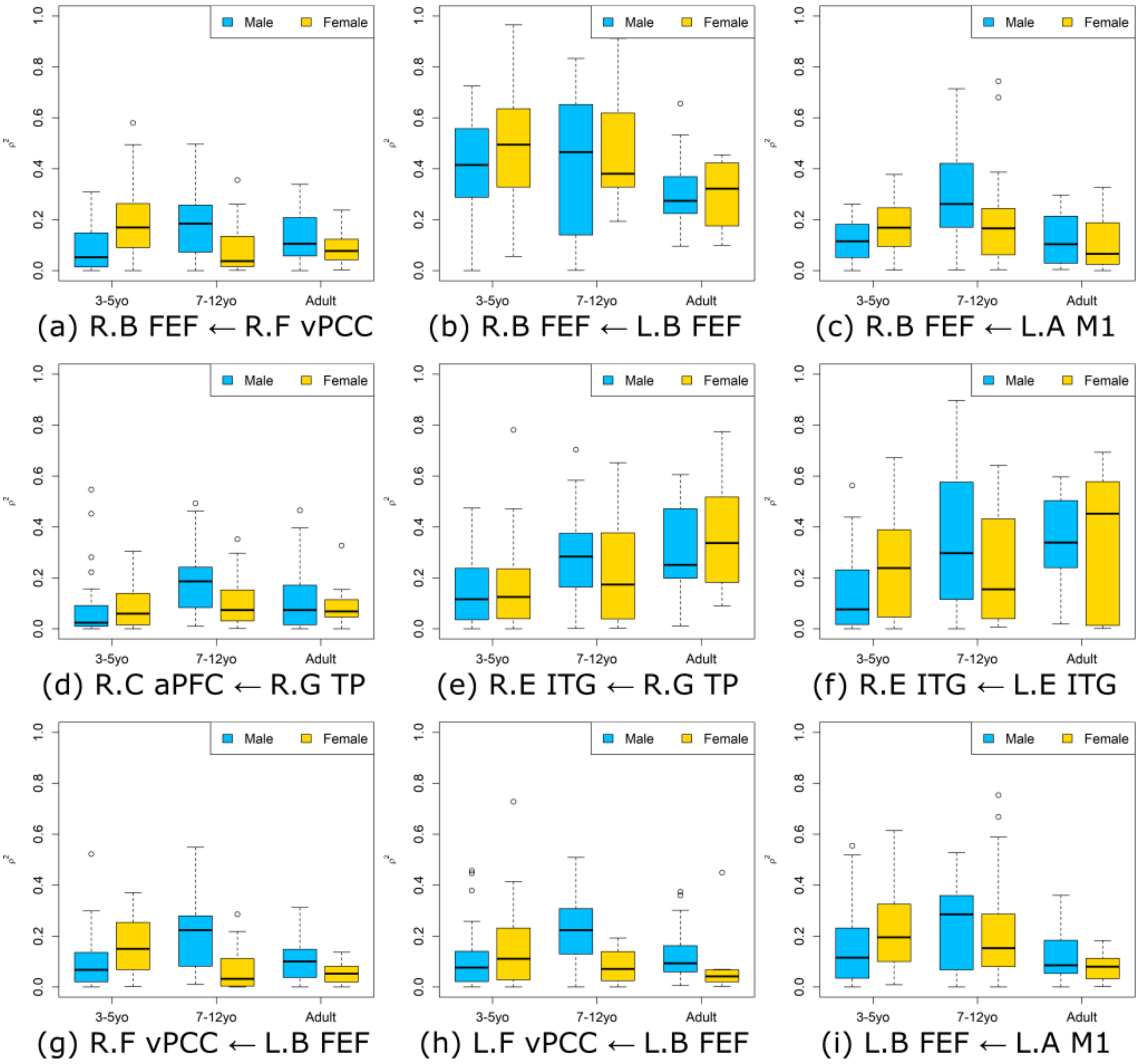

Figure S2. Boxplots for comparing distributions of $\Delta\rho^2_{U,V}$ between groups.

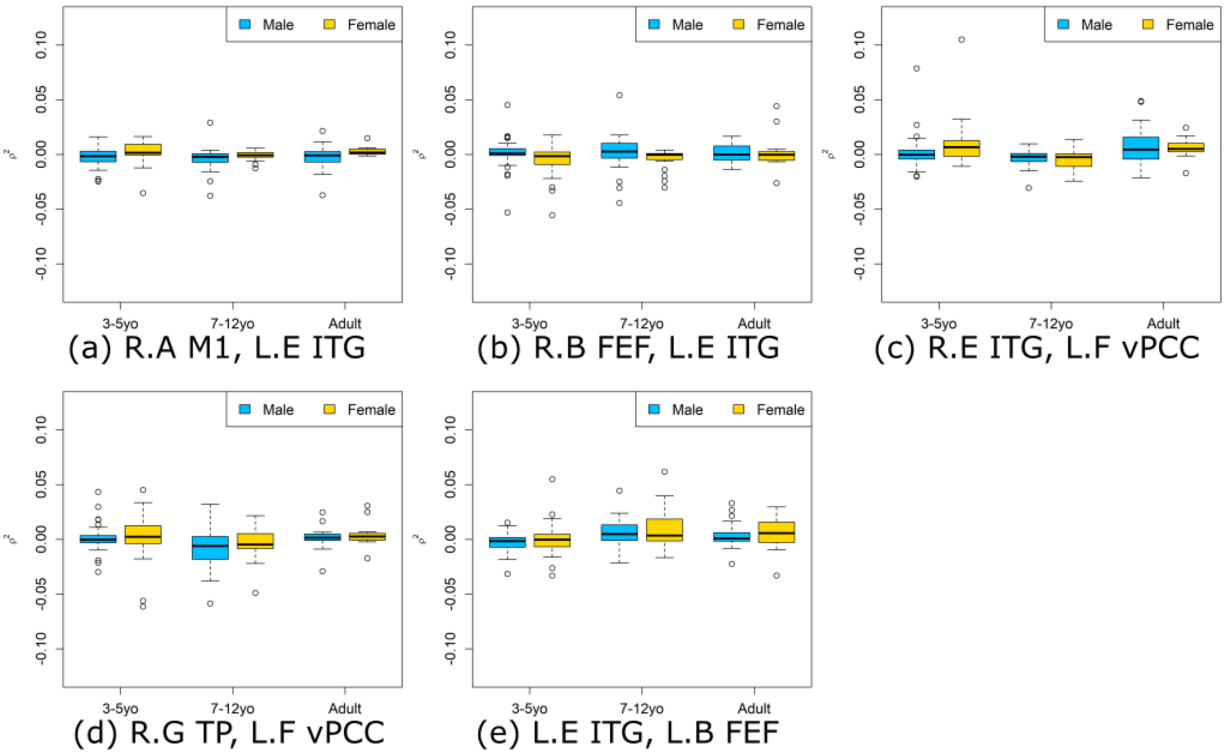

Supplement: Supplementary file 1 [file BRB3-9-e01191-s001.pdf]
